# Serotonin dysfunction in ADHD

**DOI:** 10.1186/s11689-025-09610-y

**Published:** 2025-04-22

**Authors:** Eleanor F. Jackson, Timothy B. Riley, Paul G. Overton

**Affiliations:** Department of Psychology, University of Sheffield, Western Bank, Sheffield, S10 2TN UK

**Keywords:** Attention deficit hyperactivity disorder, Serotonin, Tryptophan, Kynurenine, 5-hydroxytryptophan.

## Abstract

It is well accepted that attention deficit hyperactivity disorder (ADHD) is in part driven by dysfunction in the monoaminergic neurotransmitter system, but both the extent of dysfunction and possible therapeutic avenues presented by serotonergic neurotransmission is frequently overlooked. As such, we present key evidence for dysfunction in serotonergic transmission, as seen from biochemical, genetic and pharmacological perspectives. An overall deficit in serotonin availability is a common theme throughout the literature, thus this review aims to explore possible dysfunctions in the serotonin synthesis pathway which result in this reduced bioavailability, and investigate whether such dysfunctions could be loci of change in ADHD. We have identified several steps in transmission, namely the conversion of tryptophan to 5-hydroxytryptophan and its use of cofactor tetrahydrobiopterin, which could present promising avenues for development of novel clinical interventions for ADHD.

## Background

Attention Deficit Hyperactivity Disorder (ADHD) is one of the most common neurodevelopmental disorders, affecting an estimated 4-7.6% of children and adolescents worldwide [1–4]. The disorder is pervasive and often lifelong, with an estimated 65% of cases persisting into adulthood, or around 2.5% of the population [5, 6]. ADHD presents with clinical heterogeneity but is characterised by the triumvirate of inattention, hyperactivity and impulsivity [7]. These symptoms cause significant functional impairment throughout the lifespan. Children with ADHD often experience lower academic outcomes, peer rejection and low self-esteem, and adult symptoms can predict reduced quality of life and functional impairments [8, 9]. Furthermore, adults with ADHD have significantly lower education outcomes, employment rates, and difficulties in forming and maintaining social and romantic relationships [10–12].

Despite its ubiquity and debilitating nature, ADHD is still poorly understood in terms of its underlying neurobiology [13, 14]. As a clinically heterogeneous disorder, recent work has begun to determine whether this may be underpinned by biological heterogeneity; such lines of thought may facilitate the development of more effective, targeted interventions [15–17]. This is further supported in genetic studies for ADHD; the most recent genome-wide association study highlighted the complex aetiology of the disorder, finding 27 significant loci and 76 potential risk genes, most with moderate-to-small effects, many of which are involved in neurodevelopment and shared with other psychiatric disorders [18]. Although there are a wide range of changes within the disorder, one commonality within the literature appears to be differences within monoaminergic neurotransmission, covering dopamine, noradrenaline and serotonin (5-HT); changes to monoaminergic neurotransmitters have been robustly observed, with evidence ranging from candidate genes, neuroimaging, animal models, and most relevantly, drug action of ADHD medication [19–21]. The most commonly used ADHD medications, psychostimulants methylphenidate and amphetamine, have a diffuse range of effects which serve to increase synaptic availability of dopamine, noradrenaline and 5-HT [22, 23.^24^.^25^]. Non-stimulants used in the treatment of ADHD also increase monoaminergic signalling, either via selective noradrenergic reuptake inhibition or agonism of the alpha-2 adrenergic receptor [26, 27]. However, non-stimulant drugs like atomoxetine used in the treatment of ADHD also affect the dopamine [28] and 5-HT [26] systems.

Current ADHD medications are an effective form of symptom management for many individuals with the condition, but an estimated up to 30% of individuals do not respond to stimulant medication, and non-response to non-stimulant medication is potentially higher [29, 30]. Furthermore, stimulants have a significant risk of misuse and diversion; The USA National Survey on Drug Use and Health reported that 5.1 million people over the age of 12 had misused amphetamine or methylphenidate, approximately 1.9% of the population [31]. Long-term stimulant use may also result in a 4% increase in the risk of cardiovascular disease per year of cumulative use, with a specific risk for hypertension and arterial disease [32]. Such factors highlight the need for a greater range of therapeutics, possibly following a more symptom-based prescribing approach, looking at neurotransmitters that are not currently the primary target of ADHD medications [33]. It is accepted that altered function in a wide range of the monoaminergic neurotransmitter systems is a key biological feature of ADHD, with dopamine taking a particular focus given an increase in extracellular dopamine being a key feature of classical psychostimulant drug action; However, there are numerous members of the monoamine family of neurotransmitters, and it is unlikely dysfunction is mediated solely by dopamine [34, 35].

One potential candidate that is worthy of consideration is 5-HT, a transmitter implicated in a plethora of functions ranging from mood and appetite to the regulation of sleep [36]. 5-HT has long been overlooked and underappreciated in the field of ADHD research when compared to dopamine and noradrenaline, with some researchers doubting the involvement of 5-HT at all [37]. However, evidence for serotonergic dysfunction is growing, ranging from changes in metabolic measures to observations of candidate genes for ADHD impacting 5-HT transmission [38–40]. Arguably most importantly, serotonergic activity has also been implicated in the function of both attentional and impulsive processes, i.e. core ADHD symptoms [41–44]. Below we examine in more detail the case for the involvement of 5-HT in ADHD.

## How is serotonin implicated in ADHD?

### Biochemical and genetic evidence

Some of the most compelling evidence for 5-HT’s role in ADHD comes from measures of 5-HT in blood serum. Significantly reduced levels of serum 5-HT levels have been observed in several studies across several ADHD probands around the world [38, 45, 46]. Reductions and elevations in levels of the 5-HT precursor tryptophan and the 5-HT metabolite 5-hydroxyindoleacetic acid (5-HIAA) have also been observed in plasma, serum and urine across a range of studies, indicating a likely dysfunction in the production and catabolism of 5-HT, which will be discussed in greater detail in the 5-HTsynthesis section of this review [40, 47]. Furthermore, numerous genes involved in the synthesis and neurotransmission of 5-HT have been raised as candidate genes in the literature [48, 49]. In particular, dysfunction in the serotonin transporter gene (SERT), the protein responsible for reuptake of 5-HT in the synaptic cleft, and tryptophan hydroxylase 2 (TPH2), the enzyme which converts tryptophan to the serotonin precursor 5-hydroxytryptophan (5-HTP), have been commonly considered in such studies [50, 51]. The amount by which such genes contribute to ADHD symptoms is debated, but given the genetic complexity as evidenced by genome-wide association studies (GWAs), it is likely that ADHD is an accumulation of small-effect sizes in many genes [18].

Further interest has been shown in several of the receptors for 5-HT. Preclinical work in rodents has found knockouts for the 5-HT_1b_ receptor show increased hyperactivity and impulsivity, with impulsivity being ameliorated when 5-HT_1b_ receptors are rescued in adulthood [52–54]. Polymorphisms in 5-HT_1b_ have also been assessed in transmission disequilibrium and single nucleotide polymorphism (SNP) heritability analysis, along with the 5-HT_2a_ receptor [49, 55–57]. 5-HT_1b_ receptor SNP rs6296 has also been found to be significantly associated with high scores on the Adult ADHD Self-Report Scale (ASRS), specifically in the inattentive domain, in a candidate gene study with a moderate sample size (*N* = 990) [57]. Although genetically complex, it is evident that genetic changes to serotonergic neurotransmission are implicated in at least some cases of ADHD.

It is of particular note that alterations in serotonergic transmission have also been linked to changes in dopaminergic transmission. It is well observed that dopamine release in several brain regions is modulated by 5-HT receptor activity, and as such it is plausible that changes in dopamine and 5-HT observed in ADHD may indeed be interlinked [41, 58, 59]. For example, dopamine transporter (DAT) KO mice are often considered an ADHD model given their hyperactivity and response to psychostimulants, but such factors may be mediated by the activity of the 5-HT_1b_ receptor, since application of 5-HT_1b_ antagonists reduced hyperactivity, potentially via a regulatory mechanism [60]. Furthermore, DAT KO rodents have been found to have significantly reduced concentrations of 5-HT and its metabolites, further highlighting the complex interplay between the two neurotransmitters, and the significant likelihood that both are important to the psychopathology of ADHD [61].

### Evidence from pharmacotherapy; stimulants

Much of our understanding of the neurobiology of ADHD hinges on the mechanistic functions of the drugs which help to alleviate symptoms. Subsequently, evidence for serotonergic dysfunction can be found in the mechanisms by which ADHD medications function. The two chief medications used for the treatment of ADHD, methylphenidate and lisdexamfetamine, have a diffuse set of actions on monoaminergic neurotransmission [22], and how such actions contribute to symptomatic relief is still not fully understood. There are however a range of such actions that impact 5-HT transmission. Lisdexamfetamine, and indeed all amphetamines, are inhibitors of dopamine, noradrenaline, 5-HT and vesicular monoamine (VMAT2) transporters, although with a lesser affinity for the SERT [23]. As such, amphetamines act to increase synaptically available 5-HT, which in turn leads to greater activity at 5-HT receptors, particularly at 5-HT_1a_ receptors which act as both presynaptic inhibitory autoreceptors on serotonergic neurons and postsynaptically as heteroreceptors [24, 62]. Furthermore, methylphenidate has been shown to act as a 5-HT_1a_ agonist, thus impacting serotonergic transmission [25]. However, chronic administration of methylphenidate has been shown to downregulate the expression of the 5-HT_1a_ receptor, which has tentatively been linked to the addictive properties of psychostimulants [24, 63].

In-vitro application of d-amphetamine and methylphenidate has been found to depress sensory responses in the superior colliculus in a 5-HT-dependent manner. The effects of both d-amphetamine and methylphenidate are mimicked by application of 5-HT and application of the 5-HT antagonist metergoline blocks the effect of subsequent d-amphetamine and methylphenidate administration [64]. Such preclinical data suggested that Selective Serotonin Reuptake Inhibitors (SSRIs) may be a possible pharmacotherapy for ADHD. Indeed, a few studies have found a moderate to good improvement in ADHD symptoms, with a significant decrease in inattentive symptoms and 47% of patients showing improvement [65, 66]. Others however found no clinical effect [67], or at worst, an exacerbation of ADHD symptoms [68]. Unfortunately, there are considerable methodological problems with many of the studies that prevent clear conclusions being drawn. Firstly, a few studies have trial periods which may be too short to observe the effects of SSRI treatment; Donnelly’s study gave a 3-week administration period, whereas synaptic changes mediated by SSRIs are often not seen until 4–5 weeks post-administration [68]. It has been postulated that the activity of 5-HT_1a_ autoreceptors, which regulate the synaptic availability of 5-HT, need long periods of drug desensitisation to effectively increase the available 5-HT in ADHD trials [69]. Finally, the exacerbation of symptoms seen in Riddle’s report was observed in a sample of three children, and hence the findings are hard to generalise.

### Evidence from pharmacotherapy; alternative ADHD therapies

Non-stimulant treatments of ADHD, atomoxetine and guanfacine, also act to increase monoaminergic neurotransmitter availability; atomoxetine does this by inhibiting the noradrenaline and 5-HT transporters, guanfacine via agonism of the alpha-2 adrenergic receptor [26, 27, 70] and 5-HT receptors [71]. Despite their approval, atomoxetine and guanfacine are less efficacious than psychostimulant treatment and are not widely used [72]. In 2022, a third non-stimulant, viloxazine, was approved for use in the treatment of adult ADHD by the FDA. Viloxazine is another noradrenaline reuptake inhibitor with serotonergic activity, and has been found to elevate levels of dopamine and noradrenaline via blockade of the noradrenaline receptor, and elevates 5-HT levels in a mechanism not currently understood, highlighting the need for more preclinical investigation as to the mechanism of action of ADHD medications, particularly with regards to how 5-HT is involved [73, 74].

Preclinical and clinical work looking at drugs which target a range of serotonergic receptors have also impacted ADHD traits, implicating 5-HT as a key player in ADHD behaviours. Application of 5-HT_1a_ agonist ipsapirone, 5-HT_2a_ antagonist MDL 100907 and alpha-2 agonist GFC in Spontaneous Hypertensive Rats (SHRs), the most critically appraised rodent model of ADHD, have all been found to alleviate ADHD-like behaviours; SHRs saw a significant decrease in both hyperactive (assessed with locomotor activity in an open field test) and inattentive (using a tolerance delay and water drinking with aversive electro foot shock) behaviours [75].Furthermore, postmortem analysis of brain tissue linked this positive effect to an upregulation of 5-HT_1a_ and 5-HT_2a_ receptors alongside a downregulation of dopamine D1 receptors, suggesting an interplay between the serotonergic and dopaminergic systems in ADHD [75]. Targeting SERT, 5-HT reuptake and subsequent metabolism may also be a potential therapeutic avenue. Lamotrigine, a mood stabilizing drug that has traditionally been used in bipolar disorder, has been observed to reduce expression of SERT and thus increase 5-HT availability in vitro, which may contribute to its therapeutic effects [76]. Lamotrigine has been found to be beneficial for ADHD symptom relief in paediatric epilepsy patients and adult ADHD with comorbid mood disorders, but there has been little consideration of lamotrigine for ADHD as a sole condition [77, 78]. Combined 5-HT and noradrenaline reuptake inhibitors have also shown promise in clinical settings; venlafaxine prescribed to medication naïve ADHD adults for 6 weeks saw 75% of participants experiencing a symptom reduction of 30% or greater, alongside being found effective for ADHD treatment in children, albeit in a less efficacious manner than methylphenidate [79, 80].

Although genetic and pharmacological evidence suggests a role for 5-HT in ADHD, one of the most compelling arguments comes from the possibility that 5-HT synthesis and metabolism is dysfunctional in ADHD.

## What is typical serotonin synthesis and function?

**Fig. 1:**
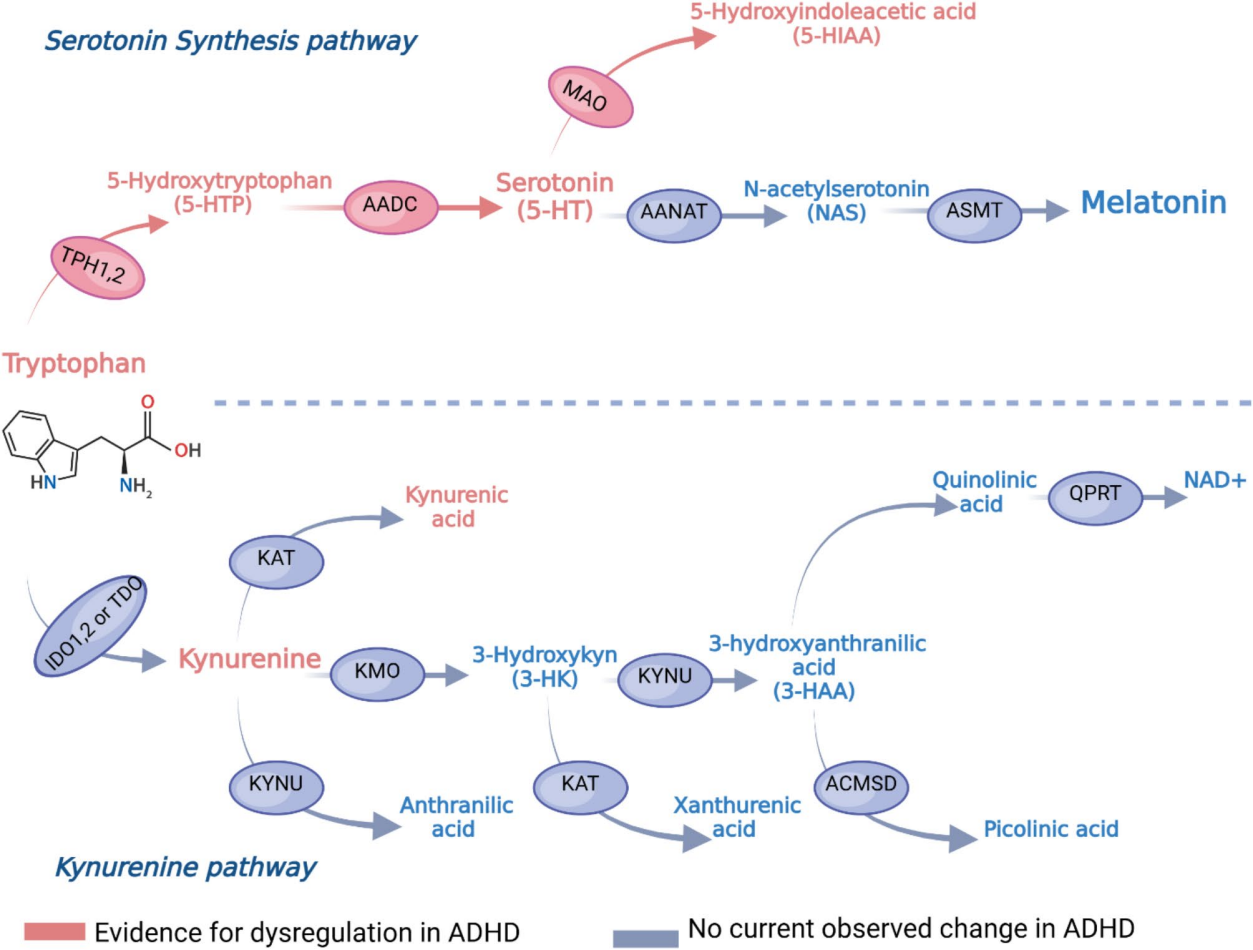
Illustrated summary of the Kynurenine and Serotonergic pathways of tryptophan metabolism, enzymes highlighted in ovals. Potential loci of dysfunction discussed in this review are highlighted in pink. Abbreviations not mentioned in text: KAT- Kynurenine aminotransferase, KYNU - Kynureninase, KMO - Kynurenine 3-monooxygenase, ACMSD - aminocarboxymuconate-semialdehyde decarboxylase, QPRT - quinolate phosphoribosyl transferase. Figure adapted from Correia and Vale [86]. Figure created using BioRender [172]

5-HT is a product of tryptophan metabolism and thus is dependent on the initial ingestion of tryptophan. Once tryptophan enters the bloodstream, it can either exist freely or be partially bound to albumin; only freely circulating tryptophan is able to pass the blood-brain barrier, meaning that there is often a variable amount of the amino acid available for synthesis [81]. Tryptophan is then hydroxylated to 5-HTP via the enzyme tryptophan hydroxylase (TPH). This is the rate-limiting step of 5-HT synthesis and requires molecular oxygen and cofactor tetrahydrobiopterin (BH4), which binds and dissociates with each turn of the reaction, to function [82, 83]. TPH exists as two isoforms, TPH1 which primarily exists in the enterochromaffin cells of the intestinal mucosa, and TPH2 which is found primarily in neurons of the raphe nuclei in the brainstem, which results in two separate pools of enteric and neural 5-HT [36, 84]. 5-HTP is then decarboxylated to form 5-HT by aromatic amino acid decarboxylase (AADC), an enzyme also implicated in dopamine synthesis [85]. Once synthesised and used, 5-HT has two metabolic fates. Primarily serotonin is converted to 5-HIAA acid by monoamine oxidases (MAO) [86]. Alternatively, serotonin in the pineal gland is converted to melatonin in a two-step process; arylalkylamine-N-acetyl transferase (AANAT) catalyses the synthesis of n-acetyl serotonin, which is then converted to melatonin using hydroxyindole-O-methyl transferase (ASMT) [87] (see Fig. 1).

The 5-HT pathway is however only one of three pathways via which tryptophan is metabolised. More than 95% of available tryptophan is metabolised via the kynurenine pathway, and a small proportion is converted by gut microbiota down the indolamine pathway [88]. The kynurenine pathway is of particular interest within ADHD, as there is evidence that there are elevated levels of kynurenine metabolites in ADHD patients [89]. Tryptophan is broken down into kynurenine by indoleamine 2,3-dioxygenase or tryptophan 2,3-dioxygenase (IDO and TDO respectively). Kynurenine is then further metabolised into a number of products, including kynurenic acid, 3-hydroxykynurenine, xanthurenic acid, 3-hydroxy anthranilic acid, picolinic acid, quinolinic acid and nicotinamide adenine dinucleotide [90, 91]. Dysregulation of the pathway could also possibly be a contributor to symptoms of ADHD; the kynurenine pathway is strongly implicated in inflammation, and metabolites such as quinolinic acid produce neurotoxic compounds such as excitotoxic N-methyl-D-aspartate agonists, with high levels being associated with anxiety and depression [92]. Other metabolites such as 3-hydroxykynurenine and 3-hydroxyanthrancillic acid have been linked to free radical formation and oxidative stress [93]. However, the metabolite kynurenic acid has a neuroprotective effect, therefore an imbalance between metabolites may in part drive dysfunction seen in conditions like ADHD [94].

## How is serotonin synthesis affected in ADHD?

### Tryptophan and uptake

When considering changes to serotonin synthesis in ADHD, it is pertinent to start at the beginning of the pathway, with tryptophan itself. Interestingly, dysfunction may occur before tryptophan even reaches the synthesis pathway; there is good evidence that ADHD patients experience differential species and quantities of bacteria in their gut microbiome, several of which may indeed either help or hinder tryptophan uptake [95, 96]. Recent research has found elevated levels of the bacterium *Allistipes putredines* in the gut flora of ADHD patients, which import tryptophan, which could reduce the amount of available tryptophan from dietary intake [97]. In contrast, further work analysing microbes and metabolites present in infant stools and cord blood found decreased levels of *Akkermansia* bacteria and increased levels of tryptophan in infants who would later develop a neurodevelopmental disorder [98]. This study focused on Autism Spectrum Condition rather than ADHD, but both conditions are neurodevelopmental disorders and have high levels of comorbidity and shared symptoms [99]. Such evidence is clearly conflicting, and microbiome research is in its infancy, but tryptophan availability as a result of the gut flora may influence ADHD development and symptoms.

Analysis of tryptophan availability in blood plasma and urine of ADHD patients has yielded mixed results; some studies report no difference between ADHD patients and controls, while others report a robust difference observable in both medicated and unmedicated ADHD patients [100, 101]. A systematic review of metabolites in ADHD found the majority of studies looking at tryptophan levels in ADHD patients found elevated levels of the amino acid [40], which suggests that deficits seen in other metabolites may occur later in the serotonin synthesis pathway, and that the conversion from tryptophan to other products may be part of the issue. Some research has looked at interventions using tryptophan in ADHD. A systematic review of such interventions found all studies used tryptophan depletion rather than supplementation, and many focused on aggression rather than core ADHD symptoms. Results reported for attention were mixed; One study found no change in scores on the test for attentional performance after tryptophan depletion, and another found an improvement in reaction times but increased omission rates for ADHD individuals in a state of acute tryptophan depletion on a modified continuous performance task [102]. A later, randomised controlled trial looking at tryptophan loading on participant attention and impulsivity found no effect on any measure of ADHD symptoms in both medicated and unmedicated groups [103]. Given this trial looked at acute administration, and results from other tryptophan studies are conflicting, it would be valuable to look at tryptophan supplementation in a larger sample with a longer administration period before ruling out the possibility of tryptophan as a therapeutic avenue.

### TPH2: Tryptophan to 5-HTP

#### Genetic evidence

Following tryptophan uptake, the next step in 5-HT synthesis is the conversion of tryptophan to 5-HTP, the rate-limiting step of 5-HT synthesis. As previously noted, this reaction is catalysed in the brain by TPH2, an enzyme of particular interest and the subject of numerous candidate gene studies, which highlight a number of risk variants [39, 41, 48, 49, 104]. It is, however, worth noting that TPH2 was not identified as a risk gene in the most comprehensive GWAs of ADHD, and several existing studies show no link between ADHD and TPH2 polymorphisms, thus evidence is still conflicting [18, 105, 106]. Familial lineage tracing of 103 families with 225 children with ADHD has found preferential transmission in ADHD probands of two alleles possessing SNPs in the upstream transcriptional control region of TPH2, the rs4570625 G allele and rs11178997 T allele [107]. Such polymorphisms have been linked to downregulation of TPH2 expression, and noted in other psychiatric disorders [108, 109]. The impact of both the rs4570625 and rs11178997 allele on response control behaviours have been assessed using EEG in a Go-NoGo task; both ADHD individuals and healthy controls in possession of the risk alleles had significantly reduced NoGo anteriorization, indicating alterations within prefrontal cortex function linked to serotonergic transmission [110].rs4570625 has also been linked to impulsive symptoms. Indiviuals in possession of the G allele in a representative birth cohort sample (*n* = 1238) had higher scores for excessive spending, giving in to cravings and ‘Insatitability by Reward’ when scored using the Reward Openness and Insatiability Scale [111]. The inverse effect has also been observed for the non-risk variant of rs4570625; individuals homozygous for the T allele in the same cohort present with lower levels of aggression, impulsivity and ADHD scores [112]. Other SNPs found in intron regions of TPH2 have been assessed in transmission disequilibrium studies and analysis of ADHD probands, and have been tentatively linked to the condition [113, 114]. Furthermore, a meta-analysis of SNPs of TPH2 has shown a small but consistent association of SNPs in non-coding regions with one or more psychopathological condition, particularly with mood disorders, a common comorbidity of ADHD [51, 115] However, when looking at TPH2 variation in ADHD, results were mixed, with the meta-analysis reporting some studies finding associations but also highlighting one large European sample study where no associations between common TPH2 variants and ADHD were observed [113]. It is also important to note that the majority of the genetic literature focuses on individuals of European ancestry, which thus impacts the generalisability of such observations.

Other TPH2 variants and their mediation of 5-HT production have been observed using rodent models. Knock-in of the rare mutant allele G1463A, an allele linked with major depression and other psychiatric disorders, found a decrease in 5-HT production of 80% when compared to wild-type, which was rescued with acute 5-HTP administration [116]. Another knock-in of human TPH2 SNP variant R439H displayed deficits in reversal learning, preservative behaviours and cognitive flexibility that could be rescued with 5-HTP or 5HT2C agonist administration, but not by methylphenidate [117]. Epigenetic modulation of TPH2 expression and subsequent 5-HT synthesis may also be a functional driver of ADHD symptoms. Observation of an adolescent cohort found a correlation between greater levels of methylation of TPH2 and higher scores on an ADHD rating scale, as well as impacted reaction time variability in Go-NoGo tasks [118]. Methylation at the CpG3 site has also been observed to impact variance in behavioural performance and neural activity in children with ADHD with the TPH2-T allele, but no such effect was observed in typically developing controls [119]. It is evident that TPH2 geneticsmay make some contribution to ADHD symptoms, presumably through the reduction in 5-HTP for conversion to 5-HT, but a full understanding of this link is still unclear. Given some of the conflicting evidence, particularly in GWAs, TPH2 dysfunction in 5-HT synthesis may be a biologically heterogeneous feature that contributes to specific ADHD symptoms, rather than a driving feature of all underlying pathology.

Interestingly, TPH1 mutations have also been linked to ADHD in association studies, particularly in offspring from maternal carriers of the mutation, although this is likely due to the reduction of maternal 5-HT and its role in neurodevelopment [120]. Furthermore, analysis of preserved cord blood in a large population sample found higher levels of cord tryptophan and 5-HTP associated with a higher likelihood of ADHD, but no change in cord 5-HT, suggesting some alteration in the tryptophan-5-HT metabolic pathway requiring greater early metabolite input, indicative of possible dysfunction early in the 5-HT synthesis pathway [121]. Exploratory analysis of variants in TPH genes in combination with variance in their regulatory 14-3-3 proteins, encoded by YWHA-genes, have highlighted a possible interplay between dysfunction in regulatory factors and TPH enzymes in driving ADHD symptoms [122]. Another possible mediating factor in both the activation of gene transcription of TPH1 and TPH2 is vitamin D. The hormone and vitamin Dmetabolite 1,25-dihydroxyvitamin D is involved in triggering a response element present in both TPH1 and TPH2 [123]. ADHD patients have indeed been observed to have lower serum levels of vitamin D, which could contribute to this initial deficit [124]. Vitamin D supplementation in RCTs also appears to improve cognitive function and behaviour in ADHD in both adults and children, however most existing trials use multivitamin protocols [125–127]. One study looking at vitamin D and magnesium supplementation in combination with vitamin D for 8 weeks found significant reductions in emotional problems, conduct problems, total difficulties and improvements in peer relations and prosocial scores [128]. Such effects may be observed as a result of more effective transcription of TPH2, and overall better functioning of enzymes involved in monoaminergic transmitter synthesis pathways.

#### Evidence from neurometabolic disorders

Another clear indication of metabolic pathways contributing to ADHD symptomology comes from neurometabolic disorders (NMDs). It is well accepted that ADHD symptoms are manifest in many NMDs, and are particularly well-described in conditions such as phenylketonuria (PKU), tyrosinemias and alkaptonuria [129]. One report found 26% of children with early treated PKU were prescribed stimulant medication for attentional dysfunction, with a correlation between plasma phenylalanine concentration and the likelihood of stimulant use [130]. Furthermore, it has been widely observed that PKU presents with reduced levels of 5-HT, similarly to that observed in ADHD [131]. Such a reduction has tentatively been linked to competition for large neutral amino acids at the blood brain barrier due to elevated levels of circulating phenylalanine [132]. It is possible that cognitive deficits and ADHD symptoms observed in PKU result from the reduction in serotonin; 5-HTP supplementation in rodent models of PKU prevented cognitive deficits in maze-learning, and 5-HTP supplementation in a case study of 2 children with PKU found mild improvements to cognitive function [133, 134]. Little more research has been conducted on 5-HTP supplementation, although one more recent study in PKU mice found post-natal 5-HTP administration improves cognitive performance in spatial and object recognition tasks, which is largely attributed to the core role serotonin plays in neurodevelopment [135].

Alternatively, the shared aspect of ADHD and neurometabolic disorders may involve the co-factor BH4, which is utlized by both TPH2 and phenylalanine hydroxylase, the enzyme responsible for converting phenylalanine to tyrosine [132, 136]. Furthermore, the BH4 co-factor plays a crucial role in the conversion of tyrosine to L-DOPA in the dopamine synthesis pathway, and in the production of noradrenaline and adrenaline subsequently synthesized from dopamine, making it crucial in the synthesis and production of many monoaminergic neurotransmitters [84]. As such, dysfunction in the synthesis of BH4 has been hypothesised as a contributing factor to both reduced serotonin and increased circulating phenylalanine in PKU, as well as contributing to psychiatric features such as ADHD in up to 6 neurometabolic disorders [137]. Sapropterin, a synthetic analogue of BH4, was approved for treatment of PKU in 2021 [138]. Supplementation with sapropterin in RCTs has been found to significantly improve ADHD symptoms in PKU individuals, and there is recent argument that sapropterin may be a suitable treatment for ADHD itself given its role in monoaminergic neurotransmitter synthesis [139, 140]. At the time of writing, there are however no trials of sapropterin for ADHD without NMDs.

### Amino acid decarboxylases: 5-HTP to 5-HT (serotonin)

The next step in serotonin synthesis is conversion of 5-HTP to 5-HT, completed with an amino acid decarboxylase. AADC’s have not been highlighted in any genetic analyses of ADHD, and have been the subject of little research [39, 49]. AADC plays an important role in the conversion of L-DOPA to dopamine as well as the conversion of 5-HTP to 5-HT, and the little work that does exist looking at AADC’s focuses on dopamine. PET imaging with fluorodopa, (L-DOPA tagged with fluorine-18 for visualisation), found significantly lower levels of fluorodopa in the prefrontal cortex, indicating overactive function of the AADC via high conversion of fluorodopa to dopamine, but no difference between other brain regions [141]. A further assessment using fluorodopa in children with ADHD found a 48% increase in fluorodopa accumulation in the midbrain when compared to controls, which would indicate hypofunction [142], contradicting the results in the prefrontal cortex. Naturally, such data cannot be fully extrapolated to AADC function in conversion of 5-HTP, and little other data exists on the nature of AADC function in ADHD. Given the lack of genetic evidence for AADC’s implication, it is likely not heavily involved in serotonergic dysfunction in ADHD.

### Post 5-HT: SERT

There is compelling evidence that the initial stage of serotonin synthesis may be impaired in ADHD, but it is also possible that disruption indeed may also occur in the later stages of metabolism, after 5-HT formation. The inactivation of 5-HT is in part regulated by 5-HT reuptake, as 5-HT that re-enters the presynaptic neuron is not metabolised and instead reused; 5-HT reuptake is mediated by SERT [143]. SLC6A4, the gene which codes for SERT, has been the subject of significant genetic research in ADHD, similar to that of TPH2 [39, 144]. There are several alleles of SLC6A4, but of particular interest are those involving the serotonin-transporter-linked-promoter region (5-HTTLPR), which is significantly implicated in the upregulation of SERT. 5-HTTLPR consists of a repeated 20–23 bp long segment, and a deletion/insertion has resulted in a long (L) and short (S) allele dependent on repeat number. There are 14 noted alleles with varying tandem number repeats; 4 short, 4 long and 4 other alleles [145]. Presence of the 5-HTTLPR L allele results in a greater transcriptional efficiency, resulting in a significantly greater amount of SERT, and thus more efficient reuptake of serotonin from the synaptic cleft and lower levels of circulating serotonin [146]. A further variation, a 17 bp variable number tandem repeat in intron 2 has two key alleles with 10 and 12 repeat units, known as Stin2.10 and 2.12 respectively, where again the longer allele has been linked to stronger transcriptional efficiency [147].

The risk of ADHD significantly increases in individuals either heterozygous or homozygous for the L variant, and meta-analysis of transmission disequilibrium tests has found a modest but significant association with Long variant 5-HTTLPR and ADHD, but no association between Stin2 variants [49, 148]. Furthermore, assessment of polymorphisms in SLC6A4 has highlighted that combinations of unfavourable alleles in this gene and the gene for adrenoceptor alpha 2a displayed a 6.15 fold increase in the risk of ADHD [149]. Behaviourally, L variant 5-HTTLPR is associated with emotional dysregulation and emotional impulsivity in ADHD adults, and childhood ADHD features in patients with other psychiatric illnesses, and may also impact the efficacy of current ADHD pharmacological interventions [150, 151]. Recent work investigating the genetic variance of SLC6A4 in a proband of Indian ADHD patients found a greater proportion of the L allele within the ADHD group, and observed significantly higher SERT mRNA expression and significantly lower 5-HIAA concentrations in the blood serum of ADHD patients [152]. One possible explanation for this is that greater serotonin uptake due to increased SERT expression leads not only to reduced 5-HT availability in the synapse, but reduced formation of downstream metabolites. There is indeed a link between low 5-HIAA levels and ADHD symptoms, with low CSF 5-HIAA concentrations being linked to increased aggression and impulsivity [153, 154]. Furthermore, alterations to SERT expression have been linked to reduced monoamine oxidase B (MAO-B) activity in ADHD; given MAO-B’s role in the catabolism of 5-HT, it may be possible that more efficacious uptake of 5-HT by SERT leads to this reduced activity [155]. MAO inhibitors, which would prevent the catabolism of 5-HT and other monoaminergic neurotransmitters, has also been deemed no more effective than placebo in ADHD treatment, which again suggests that reduced 5-HIAA and metabolites downstream of 5-HT are affected at an earlier point in the metabolic pathway, potentially due to over-efficient reuptake of 5-HT [156]. It is however unclear as to whether SERT density has a causative role in this.

### The kynurenine pathway

From the compiled evidence, it appears that any dysfunction in the serotonergic metabolic pathway in ADHD occurs in the earlier stages of synthesis. One concern with such an observation is that excess dietary tryptophan may be instead diverted to the kynurenine pathway rather than the serotonergic pathway. This has been observed in PTCHD1 knockout mouse models of ADHD, which exhibited impulsivity, poor working memory and increased levels of kynurenine and subsequent metabolites when compared to wildtype. Interestingly, administration of atomoxetine to these models ameliorated both ADHD symptoms and the increased metabolites [157].

Similar changes have been observed in humans, with a reported 48% increase in serum kynurenine and a change of 25% to the tryptophan/kynurenine ratio in ADHD individuals, with some research correlating these increases with the presentation of anxiety and depression as co-morbidities [47, 90]. However, this is not conclusive as other work has found reduced levels of tryptophan and kynurenine metabolites in the serum of individuals with ADHD or ADHD-like symptoms [158, 159]. A meta-analysis of kynurenine metabolite concentrations in serum found both tryptophan and kynurenine levels to be significantly elevated in people with ADHD, whereas kynurenic acid was significantly downregulated in ADHD, and other metabolites failed to reach significance [160]. This is particularly interesting given that kynurenic acid is widely considered to have a neuroprotective effect and may mitigate the effects of other neurotoxic metabolites such as quinolinic acid, produced later in the kynurenine pathway [161, 162]. As such, the downregulation of kynurenic acid may exacerbate the impact of downstream metabolites, even if they are not directly upregulated.

Kynurenine itself is considered neurotoxic and is involved in neuroinflammation and oxidative stress via its metabolites, the latter of which contributes to a range of psychiatric disorders [93, 163]. Research on oxidative stress in ADHD is still relatively novel, but a meta-analysis of the small number of existing studies found no evidence for decreased antioxidant activity, but modest evidence for increased oxidative stress in ADHD [164]. More recently, observation of serum kynurenine and oxidative stress markers malondialdehyde and superoxide dismutase found elevated levels of both kynurenine and the markers, suggesting elevated kynurnine synthesis could be driving oxidative stress [165]. As such, there is some consideration of whether antioxidants could be useful therapeutically in ADHD, with some such a pycnogenol, a herbal antioxidant, reducing symptoms of hyperactivity in ADHD children in preliminary investigations [166]. Co-enzyme q10 has also recently shown promising results as an add on to atomoxetine in initial non-responders, but not much research has explored q10 administration alone [167]. It is evident the role of the kynurenine pathway is multifaceted, and may cause issues beyond reducing 5-HT synthesis. However, there may be room to exploit this pathway therapeutically, or to use changes in the pathway as a biomarker.

**Fig. 2:**
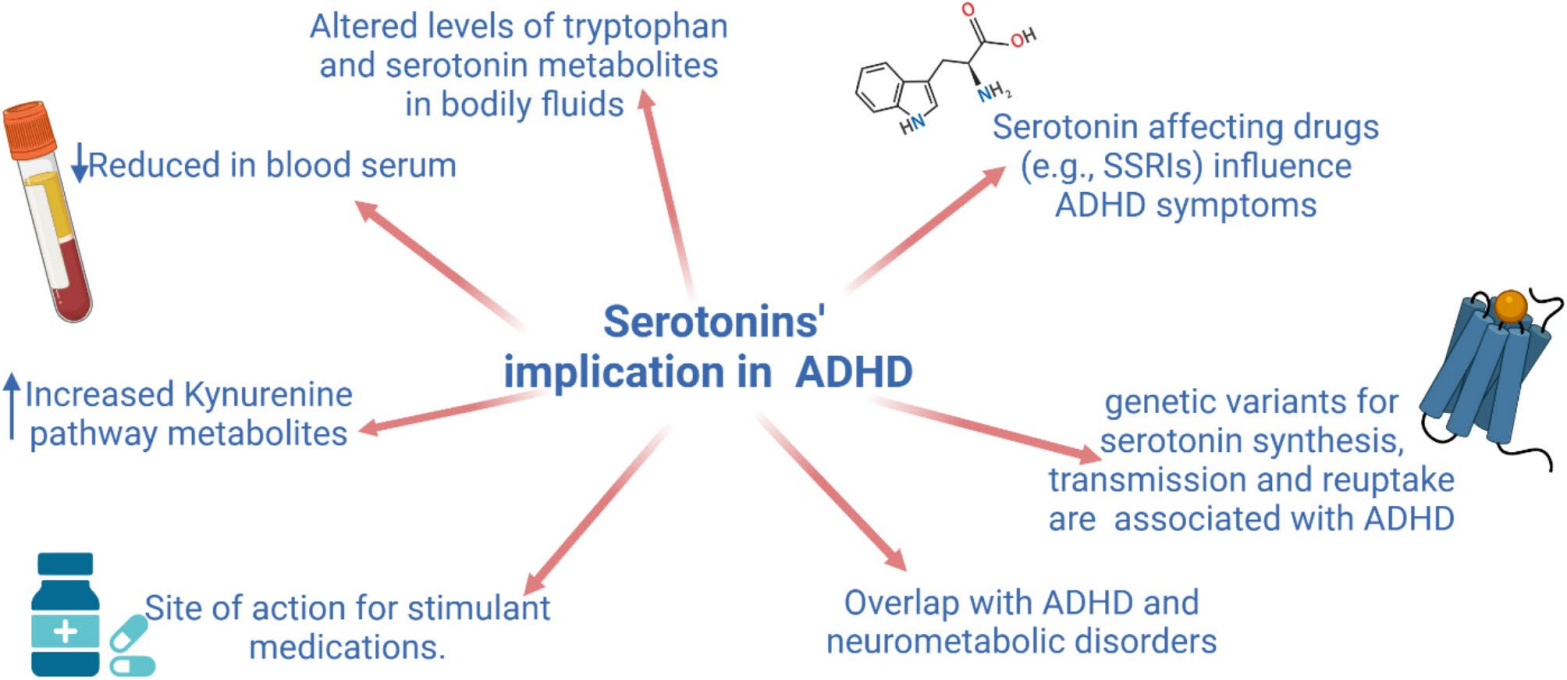
Visual summary of evidence for serotonergic dysfunction in ADHD. Graphic produced in BioRender [172]

## Conclusions

From the accumulation of research presented in the above review, it is evident there are numerous potential dysfunctional loci for 5-HT in ADHD, particularly within the context of the serotonin synthesis pathway (see Fig. 2 for summary). As a consequence, there are good grounds to widen the scope beyond the more traditional focus on dopamine and noradrenaline in ADHD. That said, this review also serves to highlight some of the limitations of current knowledge in this field; the function of AADC’s in ADHD remains largely unexplored, and there is still no full consensus on which the changes in the synthesis pathway are implicated in ADHD symptom generation. Considering the heterogeneity of the disorder, it is also possible that changes to tryptophan metabolism present heterogeneously within the disorder. As such, it seems clear that 5-HT plays a role in ADHD, but to what extent and clinical value is still unclear. Given mixed successes of various 5-HT specific pharmacotherapies, it is possible that 5-HT based intervention may be better suitable for adjunct therapies or symptom-specific treatments. Ultimately, this points to a complex picture in the regulation of tryptophan’s metabolites, which needs to be researched further to fully disentangle the various possible mechanisms of dysfunction and how these map onto ADHD symptoms.

One possible benefit that could be gleaned from understanding tryptophan metabolism is the possible emergence of biomarkers. As a disorder currently diagnosed based on reports of behaviour, there is a significant desire for ADHD biomarkers for both diagnosis and tracking medication effects [168]. Some research has also proposed a role for the kynurenine pathway as a potential biomarker [162] although research with greater sample size and diversity is needed to verify such a role. Other metabolites from the tryptophan metabolic pathways may also be of such a use. Again, this warrants a further exploration across a larger, more representative sample than reflected in the current literature, as does much of the tryptophan pathway within ADHD.

Another facet highlighted by this body of research is a range of therapeutic approaches which have not currently been explored. There is more scope to further explore the possibility of tryptophan supplementation, particularly given much of the literature looks at tryptophan depletion, and the one existing study looks at an acute rather than more chronic administration profile [104]. Further possible manipulations may come from the supplementation of 5-HTP, a nutraceutical intervention which is widely available and commonly used in traditional medicine [169]. Supplementation of 5-HTP would avoid any issues in tryptophan uptake and the rate-limiting step of serotonin synthesis, and could potentially elevate circulating serotonin levels in a way similar to that observed when using L-dopa in the treatment of Parkinson’s disease [170]. This is yet to be explored, and may impact other monoaminergic neurotransmitters beyond 5-HT given that animal modelling of 5-HTP supplementation resulted in 5-HT release from dopaminergic neurons [171]. A trial of 5-HTP supplementation and its impacts on ADHD traits however would be a good first step in considering 5-HT linked therapies, even if the impact on neurotransmission may be more complex than simple elevation of 5-HT. Sapopterin, the synthetic substitute for serotonin’s tetrahydrobiopterin cofactor, has also been shown to support reduction in ADHD symptoms in populations with neurometabolic disorders, but has not been considered for sole ADHD presentation, an area that could be explored for therapeutic benefit. Additionally, research into therapies such as antioxidants and micronutrients in restoring balance in the kynurenine arm of tryptophan metabolism are still in their infancy, giving scope for more possible therapeutic interventions. Clearly, there is much work to be done in this area, and that work could reap significant clinical benefits. The existing research poses a new, but potentially promising and exciting field whereby both advances in understanding and clinical/therapeutic options could be made.

## Data Availability

No datasets were generated or analysed during the current study.

## Abbreviations

5HT: Serotonin
5HTP: 5-hydroxytryptophan
5HTTLPR: Serotonin-transporter-linked-promoter region
AADC: Amino acid decarboxylase
AANAT: arylalkamine-N-acetyl transferase
ADHD: Attention deficit hyperactivity disorder
ASMT: hydroxyindole-O-methyl transferase
ASRS: Adult ADHD self report scale
BH4: Tetrahydrobiopterin
DAT: Dopamine transporter
GWA: Genome wide association study
IDO: Indoleamine 2,3-dioxygenase
MAO: Monoamine oxidase
NMD: Neurometabolic disorder
PKU: Phenylketonuria
RCT: Randomised controlled trial
SERT: Serotonin transporter
SHR: Spontaneous hypertensive rat
SNP: Single nucleotide polymorphism
SSRI: Selective serotonin reuptake inhibitor
TDO: Tryptophan 2,3-dioxygenase
TPH1: Tryptophan hydroxylase 1
TPH2: Tryptophan hydroxylase 2
VMAT2: Vesicular monoamine transporter
